# The Employment of Arsacetin (Ehrlich) in the Treatment of Syphilis1Deutsche Med zinische Wochenschrift, No. 35. 1908.

**Published:** 1909-05

**Authors:** A. Neisser

**Affiliations:** From the *Dermatological University Clinics*, Breslau


### The Employment of Arsacetin (Ehrlich) in the Treatment of Syphilis[1]

By A. NEISSER, M.D.

From the *Dermatological University Clinics*, Breslau.

(Translated for the Buffalo Medical Journal.)

IN connection with my urgent recommendation of a new remedy, belonging to the series of arsenic preparations, for the treatment of syphilis, it should be pointed out at once that no substitution of mercury by a new curative agent is contemplated. On the contrary, after all the experience gained on man, in the course of the last decades, especially after the numerous animal experiments which are available, we have acquired the conviction not only, but the possible assurance, that mercury is a true remedy for syphilis; a remedy capable of removing the symptoms not only, but also of destroying the virus as such. It has been reliably established that at least the syphilis of monkeys can be entirely cured by suitable treatment with mercury.

If animals which have been treated with mercury in sufficient doses—this does not involve intolerable doses, injurious in themselves—are killed, and their organs are inoculated into other animals, these organs are found to be free from poison; whereas, they are almost invariably infected in untreated animals. If such animals are reinoculated, the procedure yields the positive result

1. Deutsche Medizinische Wochenschrift, No. 35, 1908.

of a second primary lesion; whereas, untreated animals cannot be reinoculated. Briefly stated, our experience serves to show us mercury more than ever in the light of an efficient weapon for syphilis, and work of the next few years will have to determine in what way our present methods should be modified, so as to take even greater advantage of the effect of mercury than heretofore. However, we are indebted to experimental investigation for our positive knowledge of the efficiency of mercury not alone, but of the knowlege that there exist still other true curative agents for syphilis than mercury.

Today I shall limit myself to the discussion of a new preparation which contains arsenic. By means of extensive animal experimentation, Uhlenhuth and his collaborators first pointed out the protective and curative action of atoxyl in experimental dourine as well as in chicken spirillosis. On the basis of this favorable experience, Uhlenhuth, Hoffmann, and Roscher next applied atoxyl in syphilis, experimentally in animals, and therapeutically in man, giving a report of their observations. Salmon (Paris) at about the same time experimented with the arsenic treatment of syphilis. Our investigations date back to the same time, and were carried out entirely independently of those of Uhlenhuth.

In recommending at the present day these arsenic preparations for the treatment of syphilis, I refer not to experience acquired upon the human subject, but on facts established in animal experimentation. Let the results obtained with therapeutic endeavors in the case of man be ever so favorable, we know through the century long employment of mercury, how little we are able to draw conclusions from the results obtained in man, concerning the true curative action of any remedy in regard to syphilis. It is only after several decades of therapeutic use upon a large number of cases, that anything definite can be reported concerning the true therapeutic efficiency of arsenic. By means of animal experiments on the other hand, Uhlenhuth, Metschnikoff and the writer, have all shown with absolute certainty that there are preparations in the arsenic series which are capable of entirely destroying the syphilitic virus in the body without harming the animal. But these preparations of organic arsenic are actually superior to mercury, for more especially monkeys do not tolerate mercury as readily as preparations of arsenic. The results established by Uhlenhuth and his collaborators, also by Metschnikoff, were confirmed and reported by us in various essays, upon a very abundant material, in an entirely free and independent manner.

The following brief reports are herewith summarised: with acidum arsenicosum, useful results could not be obtained, in spite of large doses. It was employed subcutaneously, in all kinds of solutions, but so many disturbances occurred as the result of infiltration, such as abscess formation, and necrosis, that the carrying out of curative measures met with great difficulties. What was more serious, was the fact that it did not prove possible to guard infected animals in which treatment was begun a few days after the inoculation, against the onset of syphilis, or to cure animals which were at the time suffering from the disease. However, these experiments do not prove in any way that acidum arsenicosum is a remedy altogether unsuitable for the therapeutics of syphilis. Better results might possibly be obtained from its internal administration; for instance, experiments might be made to determine in which way it could be more readily tolerated by animals and this knowledge be used for a more continuous treatment. It might be combined with other arsenic containing preparations in a similar way as was for certain trypanosome diseases, by Laveran and Loeffler. Under certain conditions, there might accrue this advantage that each single one of the preparations be administered within readily tolerated doses, while the large dose required for a cure secured through the combination. These experiments will be discussed in detail on another occasion.

Sodium cacodylate proved entirely useless, even in very large doses. Atoxyl yielded altogether different results. I am unable to say in how far the preparation employed by us still suffers from the faults to which Hallopeau refers the unfavorable experience made with it in Germany. At any rate we are enabled to determine that the remedy, even in very large doses, was very readily tolerated by a large number of animals; and also to establish the preventive as well as the curative effect in a very considerable number of cases.

In our experiments for prevention the treatment was in part begun at once, in part the atoxyl injections not until the fifth to seventh day, in one case the thirteenth day, after the infection. The idea was to imitate the conditions as they obtain in every day practice, where a long series of days will pass before the possibility of a syphilitic infection is pointed out after a suspicious intercourse. Under a sufficient but entirely harmless dosage, by far the majority of the animals remained well, and in all these cases we convinced ourselves by a subsequent positive reinoculation, that the first inoculation had actually remained unsuccessful. An interpretation to the effect that the failure of the infection was referable to an imperfect inoculation was excluded by the large number of these experiments and by the proper control animals, which were left untreated.

Briefly stated, there can be no doubt but that atoxyl is entitled to consideration as to its preventive influence against syphilis in monkeys. Later than seven days after the inoculation, experiments were made in but one instance. Those at present under way will be reported later on. Local injections (2 c.cm. of a 2½ per cent. solution) which were applied directly under the site of inoculation, proved unsuccessful. The same failure occurred also when salicylate of mercury injections were applied in this way. The curative effect of atoxyl was tested in all stages of the disease, and positively demonstrated. It was remarked that primary lesions occasionally healed very slowly, but at last there followed a complete cure, of the local affection not alone, but also of the general disease. A cure was obtained even in those cases where the animals had been infected many months previously. Thus the latent virus was likewise destroyed.

Besides the old "atoxyl," another new preparation made by P. Ehrlich, arsacetin, was utilised and tested upon a very large number of monkeys, during my sojourn in Batavia. This preparation owes its origin to the endeavor to obtain a less poisonous but equally efficient preparation as is atoxyl. A review of the literature shows that atoxyl has an equal number of friends and of opponents among those who have employed it in men. It is pretty generally admitted that atoxyl may exert a very useful effect in the treatment of syphilis, also causing the subsidence of symptoms. But it is stated, as a rule, that toward the primary and secondary symptoms, the remedy acts weakly, at any rate, weaker than mercury, and should therefore be restricted to the treatment of tertiary syphilis, especially of malignant forms, where it often renders excellent service; also, that it affords much less protection than mercury against the onset of relapses.

A number of writers endorse its use only in malignant syphilis and in those patients in whom the employment of mercury is interfered with originally, or permanently, by an idiosyncrasy. Still others reject atoxyl altogether, on account of the severe side effects which are unfortunately not so very rare. As a matter of fact, it can not be denied that if one had to reckon with the danger of optic nerve atrophy, after a merely moderate use of atoxyl, he would hardly decide upon the employment of such a dangerous remedy; at least not until the superiority of this new remedy, as compared to the tried and tested mercury, had been demonstrated for the bulk of syphilitic cases.

How about the actual dangers of atoxyl? From the very beginning of the atoxyl treatment, the French authors led by Hallopeau, pointed out that there must be a difference between the atoxyl preparations made and used in Germany and France. As a matter of fact, all the said cases of optic nerve atrophy, and of the other side effects upon the stomach and bowel, kidney, and the like, a very much greater number have been noted under employment of the old German atoxyl, whereas the French preparation proved to be less poisonous. Hence, there either must have existed toxic side substances in the German atoxyl from the start, or they must have formed in it under improper preservation of the solution. This idea suggests itself even more because atoxyl solutions have long been known to decompose easily, to change in color (turn yellow), and to become a possible source of danger because they can not be simply sterilised by boiling, for the reason that boiling leads to chemical changes.

It is only very recently, as far as I am informed, that the mode of manufacture has been changed in Germany, so that at present an absolutely pure crystallised preparation is put upon the market. It is to be hoped that in consequence, and also through caution with the dosage, the serious side effects of the past will not be repeated. Doses of 6.0 to 6.5 grms. (90 to 97½ grains) had been stated, especially by Lesser, as the maximum for a total treatment. Robert Koch, on his expedition, showed that infinitely larger amounts of atoxyl may be employed. Blindness did not occur when he employed atoxyl in doses of 0.5 grm. (7½ grains) each, twice weekly, for months together, but it is true that individual doses of 1.0 grm. (15 grains) did induce blindness.

Accordingly, we were confronted on the one hand with the fact, as crystallised by animal experimentation, that atoxyl is a curative agent against syphilis, when the treatment is liberal enough; on the other hand, it had to be admitted that the dose required to reach this goal might not prove beyond objection in man, so that a thorough atoxyl treatment would meet with considerable difficulties, on account of the poisonous properties of the preparation.

In this state of affairs, Ehrlich recommended a new atoxyl preparation. In the first place, he showed that the former chemical interpretation of atoxyl as a "meta arsenate anilid" was an erroneous one. He proved together with Bertheim, that atoxyl has an entirely different constitution, and that it is the mono-sodium-salt of p-amino-phenyl-arsenate. From this fundamental substance, the acetyl compound is prepared, the sodium salt of which represents sodium acetylparamidophenylarsenate, for which the abbreviation "Arsacetin" will be used in what follows. In contradistinction to the parent substance, the solutions of arsacetin are extremely stable towards high temperatures and may even be heated in the autoclave to 130° C. without undergoing the splitting off of arsenic acid. I have been working with this arsacetin for many months in Batavia, and have established the following conclusions:

1. The preparation is certainly less poisonous than old atoxyl, always keeping in mind, of course, that 0.6 grm. (9 grains) of the new preparation equal 0.5 grm. (7½ grains) of the old. Healthy as well as diseased animals tolerated very much larger doses of arsacetin than of atoxyl.

2. The curative effect upon syphilis was at least equal to that of the old preparation. I even believe the curative action to be greater than with old atoxyl, as far as this is demonstrable.

3. No decomposition of any kind could be demonstrated in the solutions themselves, after they had been kept for a long time, especially was there no yellow discoloration. Also in animals, there appears no difference in the toxic effect of very old, or of newly prepared solutions; the solution was not even changed in any way by daily boiling.

Corresponding to these experiences made on animals, our syphilitic patients have been treated for the better part for several months with this new arsacetin, which I feel able to recommend as a very useful preparation. Regarding its therapeutic efficiency, similar statements must be made as those applied above to atoxyl. In very numerous cases, it promptly removes existing symptoms, in others it fails. Relapses are not prevented, and even appear in the course of the treatment. Concerning the control of the symptoms, it is inferior to mercury. But I must insist once again that the attitude of judging the efficiency of an antisyphilitic agent by its symptomatic curative action, appears to me an entirely erroneous one, when proof has already been furnished, in animal experimentation, that the remedy possesses a positive, curative, virus destroying action.

I consider it our duty to utilise these experiences gained on animals for the treatment of human syphilitics. If at present we are unable to prove positively that human syphilis is cured more rapidly and reliably by these new preparations than heretofore, on the other hand no one is justified in doubting it. I would regard it as wrong, however. to limit the treatment to these atoxyl preparations and to abandon the positively tested effect of mercury. Meanwhile, there is no necessity at all to decide in favor of one or the other remedy, as the two may be employed side by side. This can be done so much more readily as it has been shown, in many trypanosome diseases not only but also by ourselves in case of syphilis, that combinations of mercury with the new arsenic preparations render excellent services. It seems as if one might really remain within smaller limits of dosage for each remedy, when the two are combined,—meaning limits such as must be designated as entirely harmless,—unless there exists an idiosyncrasy, without diminishing the therapeutic effect.

Concerning the side effects of arsacetin, we have not observed a single instance of optic nerve atrophy or a similar damage to the nervous system, although we can look back upon a large number of treatments. Neither have disturbances of the kidney been noted. More frequently, but far more rarely than under employment of atoxyl, we observed temporary pains in the stomach and bowel, which in very rare cases increased to vomiting. It was a noteworthy fact that the vast majority of these disturbances occurred in women; in men, they were positively exceptional. Recently these disturbances have been controlled with apparent success by the administration of magnesia usta, and they have been guarded against by giving a small teaspoonful of the magnesia shortly before the injection and again four or five hours after it. other alkaline salts seem to act in a similar manner, at least one of my patients who was taking the Karlsbad cure, found that the injections which otherwise caused a certain amount of distress in his stomach and bowels, agreed with him much better while he was using the alkaline waters.

Sudden elevations of temperature, rapidly subsiding again, were noted a few times, without being associated with other signs of intoxication. In a general way, it may be said that in the vast majority of the cases, there occurred no disturbances at all, and when this was the case, they were but insignificant. Nevertheless, it is advisable, for the present at least, to observe certain precautions when making use of this treatment, by giving in every case a small initial dose to start with, 5 to 10 cgrm., and by not treating all syphilitics unconditionally with arsacetin, but only those in whom no parenchymatous organic disturbances can be demonstrated. Although the innumerable animal experiments which have already been performed with arsacetin, have shown that the toxicity—more particularly that referable to arsenic—is relatively very slight, and that perfectly enormous doses (as calculated for man) are required to produce a fatal outcome, or degenerative organic changes; and although arsacetin is readily tolerated by individuals with healthy organs, it would seem as if diseased or weakened organs are more susceptible to the toxic influence. Individuals, for instance, who are suffering from myocarditis, are preferably not subjected to this mode of treatment at all.

In nephritic individuals, the same procedure is indicated as in treatment with mercury. In the first place, the exact condition of the patient must be determined by a prolonged period of observations, as to the degree of the albuminuria, the kind and number of cylinders, cells, and the like. Then the tolerance of the preparation by the kidneys must be carefully studied by the administration of small daily doses. It will be found that no aggravation of the kidney trouble, in any direction whatsoever, occurs in a very large number of cases. Concerning the mercury cures, I can positively assert that a very essential improvement is obtained in many cases, perhaps allowing the conclusion that the renal affection in these cases was caused by syphilis.

Patients having tabes, paralysis, severe neurasthenia, and the like, occasionally present attacks of vertigo or aggravation of the nervous symptoms, and the treatment was therefore invariably interrupted in these cases as soon as such disturbances became manifest. Waterman a year ago recommended in case of atoxyl that it be omitted in syphilis of the brain as well as in tabetic atrophy of the optic nerve, especially as he was unable to obtain favorable results in the treatment of central affections of the optic nerve on a syphilitic basis. But in this connection, we may rightfully inquire —(I here accept an opinion expressed by O. Foerster)—if it is not precisely the aggravation of the symptoms in tabetics, for instance, which indicates that an action of the remedy upon the pathological foci exists, and that the treatment should therefore be kept in those cases in which certain nervous symptoms are increased during the treatment, perhaps through the treatment. It is probably correct, however, as I believe in conformity with Ehrlich—to diminish the doses in these cases, to such a degree that the reactions are reduced to a slight measure of severity.

Our treatments were carried out in the following manner: based upon the experience of Robert Koch, I injected 0.6 grm. (9 grains), arsacetin (0.5 grm. atoxyl (7½ grains), on two successive days of each week. We have injected occasionally 0.75 grm. (12 grains), and reduced the five days' interval between the two injection days to four days in certain instances.

It cannot yet be decided whether or not it is absolutely necessary to administer the injections on two successive days, or if the same result is obtained by injecting regularly, every fourth day, for instance. In analogy with the experience gained in trypanosome infections, it would seem advisable for the time being to give as large doses as possible, in a short space of time, rather than at longer intervals. Such injections of 0.6 up to 0.75 grm. (9 to 12 grains), were not carried out by me for a longer time than ten weeks, equalling a consumption of 12.0 to 14.0 grm. (180 to 210 grains) pro treatment; invariably, I wish to repeat, without any serious side effects.

The preparation was employed in either a 10 or 15 per cent. solution. The latter affords the advantage that it is not necessary to inject very large quantities of fluid. The transitory pain at the site of the injection is thus still more diminished. But there is this drawback that in the 15 per cent. solutions. the salt is precipitated by cold, so that the fluid must be treated before each injection.

Infiltrations or abscesses were not observed in a single instance, for the solutions are easily sterilised by repeated boiling. In the majority of the cases, a combination of mercury with arsacetin was made use of for the time being. Either inunction cures were prescribed (for patients in the clinics, or those leading a very domestic. life), or injections were made with the new gray oil, or a calomel ointment 40 per cent. These two preparations I am able to recommend very strongly as producing far slighter symptoms of irritation than our old preparations. However, a very special manufacture is required, such as is guaranteed pure.

The mercury and arsacetin treatments were carried out together in the past. Perhaps they might be applied alternately also. Neither is there apparently any objection to the internal administration of arsacetin. For the time being, I have not exceeded the dose of 0.12 grm. (2 grains) daily and therefore am unable to say up to what doses we may go in the internal administration of the remedy. Again, the question arises if the daily administration of relatively small amounts is really just as efficient as single very large doses. I am at present engaged upon animal experiments, for the elucidation of this point.

The combination of various arsenic preparations, notably atoxyl and acidum arsenicosum, recommended by Loeffler has been already pointed out above. The objection to the effect that this constitutes merely the simultaneous employment of two arsenic preparations, must be refuted from the start. Ehrlich's investigations have rendered it probable that under employment of atoxyl, or arsacetin, the destruction of the parasites is referable in the first place to the products originating through reduction, the arsen-oxyd and arsen substances; whereas, a secondary part is played by the constituents liberated by splitting,—anilin and inorganic arsenic acid,—provided these play any part at all.

It was also shown by Ehrlich through his experiments upon trypanosomes rendered immune to the toxin, that different preparations, although they all contain arsenic, may unfold entirely distinct therapeutic activities. A trypanosome culture which is immune against an arsenic containing preparation A, is not immune against an arsenic preparation B, which is apparently very closely related to preparation A.

Whether it will be possible to transfer at once to human therapeutics the combinations recommended by Loeffler, Laveran, and others—is still a matter of speculation. It is true that in animals, very favorable results have been obtained by the combination of arsenic acid with atoxyl, but in these cases the doses were such as cannot be employed in man, with the figures as calculated for the human subject. Especial attention is called to the following fact: the side effects observed in man are due to impure atoxyl, having contained arsenic acid; and since it must be assumed that the amounts of arsenic were relatively inconsiderable, but nevertheless sufficient to cause the injurious side effect, it results that in the case of the human subject, inorganic arsenic acid must be regarded as an especially dangerous substance, in certain individuals. Meanwhile, it is presumably a fact that arsenic acid, administered internally, is transformed in part into arsenious acid, which acts much more strongly in man; hence, it is to be feared that by combining the pure atoxyl preparations with arsenic acid or arsenious acid, those dangers are encountered which were to be avoided by the purification of the preparations. (Compare Ehrlich).

Prolonged research in this connection, is evidently required before all these points can be determined. This labor will necessarily fall to the lot of scientific institutions and experimental investigations. Again it must be repeated that in the case of a chronic disease such as syphilis, the experiences and observations made on the patients, favorable as well as unfavorable, do not at once permit a conclusion as to the actual value of any remedy or method. In the treatment of patients all that can be established for the time being is the effect upon the symptoms, whereas. nothing at all can be stated concerning the actual eradication of the disease.

However, as we are not in any way justified in doubting this possibility of obtaining the actual eradication of the disease, in the case of man, and this more rapidly and reliably when the new preparation is utilised besides the good old remedy, mercury—it becomes our duty, I am convinced to take advantage of this possibility. It should not be forgotten what prolonged labors and constantly renewed modifications were required until we had learned to recognise in full the curative action of mercury and to utilise all its therapeutic power.

When all is said and done, arsacetin is merely the first step on the road toward new and improved antisyphilitic agents. At the Frankfurt Congress of the German Dermatological Society, the principles for the continuation of these investigations were extensively discussed by Ehrlich, who also reported at the same time certain reduction products of other organic arsenic preparation, which were of extraordinary efficiency against trypanasomes in animal experiments and proved vastly superior to arsacetin and atoxyl. Ehrlich concluded his address by saying that even if this substance should prove in the end to be unsuitable for man, we must not allow ourselves to give up and abandon all hope in the search for improved remedies. We must simply keep on along the path which is now clearly outlined for us. As a matter of fact, I have no doubt whatsoever, but that this goal, already reached by Ehrlich in the treatment of dourine, will also be attained by us in the treatment of syphilis.

